# Supplementary material for: Lysyl oxidase drives ccRCC progression by coordinating HIF-2α transcription program with tumor microenvironment
Source: bioRxiv. 2026 Jul 27:2026.07.24.739825. Preprint. [Version 1] doi: 10.64898/2026.07.24.739825 (PMC13456076; doi:10.64898/2026.07.24.739825)
Supplement: Supplementary file 1 [file NIHPP2026.07.24.739825v1-supplement-1.pdf]

bioRxiv preprint doi: https://doi.org/10.64898/2026.07.24.739825; this version posted July 27, 2026. The copyright holder for this preprint (which was not certified by peer review) is the author/funder, who has granted bioRxiv a license to display the preprint in perpetuity. It is made available under a CC-BY 4.0 International license.

Supplementary Material

Lysyl oxidase drives ccRCC progression by coordinating HIF-2α transcription program with tumor microenvironment

Burge Ulukan[1], Ozge Saatci[1], Ariel Madrigal[2, 3], Minjun Kim[2, 3], Wensheng Tian[4], Mustafa Soytas[3,5,6], Zohreh Mehrjoo[2, 3], Ozlem Sener Sahin[1], Kukkamadi Sreenivas[7], Chintada Nageswara Rao[7], Tamiko Nishimura[3], Virginie Pillon[8], Jean-Sebastian Anoma[9], Elizabeth Hill[9], Janusz Rak[10 11], Campbell McInnes[7], Morag Park[8, 12, 13], Fadi Brimo[14], Simon Tanguay[5], Ryan Charles Russell[4, 15, 16], Hamed Najafabadi[2, 3], Yasser Riazalhosseini[2, 3, *], and Ozgur Sahin[1, *]

[1]Department of Biochemistry and Molecular Biology, Hollings Cancer Center, Medical University of South Carolina, Charleston, SC, 29425, USA

[2]Department of Human Genetics, McGill University, Montreal, QC, H3A 0G1, Canada

[3]Victor P. Dahdaleh Institute of Genomic Medicine, Montreal, QC, H3A 0G1, Canada

[4]Department of Cellular and Molecular Medicine, University of Ottawa, Ottawa, ON, K1H 8M5, Canada

[5]Division of Urology and Uro-oncology, McGill University. Montreal, QC, H4A 0B1, Canada

[6]Department of Urology, Faculty of Medicine, Istanbul Medipol University, Istanbul, Turkey

[7]Department of Drug Discovery and Biomedical Sciences, University of South Carolina, Columbia, SC 29208, USA

[8]Rosalind and Morris Goodman Cancer Institute, McGill University, Montreal, QC, H3A 1A3, Canada

[9]Department of Public Health Sciences, Medical University of South Carolina, Charleston, SC, 29425, USA

bioRxiv preprint doi: https://doi.org/10.64898/2026.07.24.739825; this version posted July 27, 2026. The copyright holder for this preprint (which was not certified by peer review) is the author/funder, who has granted bioRxiv a license to display the preprint in perpetuity. It is made available under a CC-BY 4.0 International license.

[10]Research Institute of the McGill University Health Centre, Montreal, QC H4A 3J1, Canada

[11]Department of Pediatrics, McGill University, Montreal, QC H4A 3J1, Canada

[12]Department of Biochemistry, McGill University, Montreal, QC, H3G 1Y6, Canada

[13]Department of Oncology, McGill University, Montreal, QC, H3A 0G4, Canada

[14]Department of Pathology, McGill University Health Centre, Montreal, QC, H4A 3J1, Canada

[15]Ottawa Institute of Systems Biology, University of Ottawa, Ottawa, ON, K1H 8M5, Canada

[16]Immunity and Inflammation, University of Ottawa Centre for Infection, Ottawa, ON, K1H 8M5, Canada

[*]**Correspondence to** sahin@musc.edu or yasser.riazalhosseini@mcgill.ca

Keywords: clear cell renal cell carcinoma, lysyl oxidase, single-cell transcriptomics, pseudohypoxia, HIF-2α proteostasis, angiogenesis, tumor microenvironment, therapeutic resistance

bioRxiv preprint doi: https://doi.org/10.64898/2026.07.24.739825; this version posted July 27, 2026. The copyright holder for this preprint (which was not certified by peer review) is the author/funder, who has granted bioRxiv a license to display the preprint in perpetuity. It is made available under a CC-BY 4.0 International license.

SUPPLEMENTARY METHODS

Immunoblotting

Protein isolation and immunoblotting were performed as previously described[1]. Briefly, RIPA buffer was used to isolate total protein lysate in the presence of protease and phosphatase inhibitor cocktails, and protein concentrations were measured using the BCA Protein Assay Reagent Kit (Thermo Fisher Scientific, Cat. no. 23223, 23224). Equal amounts of protein were separated on 8-10% SDS-PAGE gel. Separated proteins were transferred onto PVDF membranes (Bio-Rad, Cat. no.1620177) using a Trans-Blot turbo transfer system (Bio-Rad) and incubated with primary antibodies at dilutions indicated in **Supplementary Table 2**. Horseradish peroxidase-conjugated anti-mouse or anti-rabbit antibodies (**Supplementary Table 2**) were used as secondary antibodies, and signals were detected by enhanced chemiluminescence (Thermo Fisher Scientific, Cat. no. A38556). Images were acquired using Image Lab Software (Biorad) or iBright Software (Invitrogen).

Quantitative RT-PCR (qRT-PCR) analysis

Total RNA was extracted from cultured cells using Quick-RNA Miniprep Kit (Zymo, Cat. no. R1055), and cDNAs were generated using RevertAid RT Reverse Transcription Kit (Thermo Fisher Scientific, Cat. no. K1622). qRT-PCR analysis was performed with gene-specific primers, using LightCycler 480 SYBR Green I Master kit (Roche). *HPRT1* and *ACTB* were used as housekeeping genes. The average Ct value was calculated from triplicates of each sample, and the relative mRNA expression was determined. Sequences of the qRT-PCR primers are listed in **Supplementary Table 3**.

bioRxiv preprint doi: https://doi.org/10.64898/2026.07.24.739825; this version posted July 27, 2026. The copyright holder for this preprint (which was not certified by peer review) is the author/funder, who has granted bioRxiv a license to display the preprint in perpetuity. It is made available under a CC-BY 4.0 International license.

Transient transfection with siRNAs and overexpression reporters

Transfections were carried out by seeding cells in media containing no antibiotics and changing media with Opti-MEM reduced serum medium (Thermo Fisher Scientific, Cat. no.31985070) prior to transfection with Lipofectamine 2000 (Thermo Fisher Scientific, Cat. no.11668019) in 6-well or 96-well format. siRNA transfections were performed at a final concentration of 100 nM (**Supplementary Table 4**), and RNA or protein was isolated after 48 hours of transfection. Overexpression plasmids were transfected at 500 ng per well in 6-well plates.

Co-immunoprecipitation

Cells were treated with LXG6403 (5, 15, and 30 µM) for 4 hours and lysed in lysis buffer (50 mM Tris-HCl pH 7.0, 150 mM NaCl, 0.2% NP40, 7.5% glycerol, protease and phosphatase inhibitor cocktails. Lysates were clarified by centrifugation, and 1 mg of total protein per condition was incubated overnight at +4 ºC with Dynabeads Protein G (Thermo Fisher Scientific, Cat. no.10003D) pre-coated with the indicated antibodies (**Supplementary Table 2**). Beads were washed and resuspended in 60 µL 1X SDS sample buffer, boiled at 70 ºC for 10 min, and loaded onto a polyacrylamide gel. Immunoblot was done using antibodies provided in **Supplementary Table 2**.

Chromatin immunoprecipitation (ChIP) assay

ChIP assays were performed as described previously[2]. Briefly, 786-O cells that were grown to 70% confluency were crosslinked with 1% formaldehyde for 10 min, followed by quenching with 125 mM glycine for 5 min. Cells were lysed in 500 µL lysis buffer, and nuclear lysates were extracted.

bioRxiv preprint doi: https://doi.org/10.64898/2026.07.24.739825; this version posted July 27, 2026. The copyright holder for this preprint (which was not certified by peer review) is the author/funder, who has granted bioRxiv a license to display the preprint in perpetuity. It is made available under a CC-BY 4.0 International license.

Sheared chromatin was incubated overnight at 4 °C with HIF-2α antibody and magnetic beads with slow agitation (**Supplementary Table 2**). IgG antibody was used as the negative control. Samples were washed with low and high salt wash buffers, and Proteinase K treatment was done for 2 h at 62 °C with shaking. Samples were incubated at 95 °C for 10 min and separated from the beads using a magnetic separator. DNA was isolated, and ChIP-qPCR was performed using primers targeting the VEGFA, SERPINE1, PTHrP, and CCND1 promoters. The results were normalized to the bead-only control and represented as fold enrichment. Sequences of the ChIP-qPCR primers are listed in **Supplementary Table 5**.

Lysyl oxidase activity

Lysyl oxidase activity assay within cells or within tumors was measured using the fluorometric Lysyl Oxidase Activity Assay Kit (Abcam, Cat. no. ab112139) according to the manufacturer's instructions. Briefly, serially diluted replicates of tumor lysates or cell supernatants were incubated with reagent mix and subsequently measured in a fluorescence microplate reader at Ex/Em = 540/590 nm wavelength. Tumor lysates were prepared by sonicating tissue samples in extraction buffer (6 M urea, 10 mM Tris, pH 7.4, and protease inhibitors) followed by centrifugation.

Cell fractionation and chromatin extraction

Cytoplasmic and nuclear protein fractions were isolated using the Nuclear Extract Kit (Active Motif, Cat. no.54001) according to the manufacturer's protocol. Cells were lysed in a hypotonic buffer to release cytoplasmic content, followed by high-salt extraction of nuclear proteins. Fraction purity was validated by immunoblotting with cytoplasmic marker α-tubulin and nuclear marker lamin B1 (**Supplementary Table 2**). Resulting fractions were followed by immunoprecipitation.

bioRxiv preprint doi: https://doi.org/10.64898/2026.07.24.739825; this version posted July 27, 2026. The copyright holder for this preprint (which was not certified by peer review) is the author/funder, who has granted bioRxiv a license to display the preprint in perpetuity. It is made available under a CC-BY 4.0 International license.

For chromatin fractionation, cells were lysed using buffer (10 mM Tris-HCl pH 7.5, 10 mM NaCl, 3 mM $MgCl_2$, 0.5% NP-40, with protease/phosphatase inhibitors) and incubated for 45 min on ice. Lysates were centrifuged at 16,000 × g for 15 min at 4°C to separate the cytosolic fraction (supernatant). The pellet was washed once with 200 μL wash buffer (10 mM Tris-HCl pH 7.5, 10 mM NaCl, 3 mM $MgCl_2$, 1 mM EDTA), centrifuged, then resuspended in 200 μL wash buffer and sonicated (3 × 10 sec pulses). This chromatin fraction was quantified by BCA and analyzed by Western blot, loading 2× protein amounts compared to cytosol. Histone H3 and α-tubulin were used as fraction controls.

Tubule Formation and ex vivo angiogenesis assays

HUVECs were labeled with 2 μM calcein-AM (Invitrogen) for 30 min at 37°C prior to cell plating. Labeled cells (2×10^4) were seeded on growth factor-reduced Matrigel-coated 96-well plates and cultured in endothelial growth medium (Lonza, Cat. no.CC3162) containing axitinib (1 μM; MedChemExpress, Cat. no. HY-10065), the LOX inhibitor LXG6403 (5 μM), or conditioned media derived from LOX-modulated HEK293T cells. Tube formation was assessed after 16-18 h of incubation. Fluorescent images were captured using an inverted fluorescence microscope, and tube length, branching points, and network area were quantified using the Angiogenesis Analyzer plugin in ImageJ[3].

Thoracic aortas were isolated from non-tumor-bearing C57BL/6 mice (The Jackson Laboratory; RRID:IMSR_JAX:000664) and sectioned into 1-mm pieces. Aortic explants were embedded in growth factor-reduced Matrigel (Corning, Cat. no.356231) and cultured in endothelial growth medium (Lonza, Cat. no.CC3162) containing vehicle, LXG6403, or axitinib

bioRxiv preprint doi: https://doi.org/10.64898/2026.07.24.739825; this version posted July 27, 2026. The copyright holder for this preprint (which was not certified by peer review) is the author/funder, who has granted bioRxiv a license to display the preprint in perpetuity. It is made available under a CC-BY 4.0 International license.

for 3 days. Microvessel sprouting was imaged using brightfield microscopy, and sprout formation was quantified using ImageJ software.

Colony formation, sphere assays, and 3D cultures

For colony formation, cells (1,000 cells/well) were seeded in 12-well plates and treated with the indicated inhibitors for 14-21 days. Colonies were fixed, stained with 0.2% crystal violet, and quantified at the experiment endpoint. Spheroid assays were performed using ultra-low attachment plates in serum-free DMEM/F12 (Thermo Fisher Scientific, Cat. no.11330032) supplemented with B27 (Thermo Fisher Scientific, Cat. no.17504044) EGF (20 ng/ml, Peprotech, Cat. no.100-15), and bFGF (20 ng/ml, Peprotech, Cat. no.100-26, 100-19). Sphere size and number were quantified after 14-21 days. Collagen-embedded 3D culture assays were performed as previously described[4]. Briefly, neutralized collagen matrices were prepared using rat tail collagen I (Corning, Cat. no.354236) at a final concentration of 1 mg/mL with pH adjustment using 1 N NaOH. Cell pellets were resuspended in the collagen mixture prepared in PBS and seeded into 96-well plates. Collagen matrices were allowed to solidify for 1 h at room temperature prior to the addition of culture media. Embedded cells were subsequently cultured at 37 °C for the indicated time periods, and drug treatments were initiated 12 h after seeding. Cell viability was assessed using the CellTiter-Glo 3D assay (Promega, Cat. no. G9683) according to the manufacturer's instructions.

Migration and invasion assays

Transwell migration and matrigel invasion assays were performed using 8-µm pore size inserts (Corning, Cat. no.3428). Cells were plated in serum-free medium, allowed to migrate for 24 h

bioRxiv preprint doi: https://doi.org/10.64898/2026.07.24.739825; this version posted July 27, 2026. The copyright holder for this preprint (which was not certified by peer review) is the author/funder, who has granted bioRxiv a license to display the preprint in perpetuity. It is made available under a CC-BY 4.0 International license.

(migration) or 48 h (invasion), then fixed with 4% PFA, stained with 0.2% crystal violet, and quantified using Image J.

Angiogenesis protein array

Serum angiogenic profiling was performed using the Proteome Profiler Mouse Angiogenesis Array Kit (R&D Systems, Cat. no. ARY015) according to the manufacturer's instructions. Briefly, sera collected from treated mice were incubated with nitrocellulose membranes pre-spotted with angiogenesis-related capture antibodies. Membranes were processed following the manufacturer's protocol, and images were acquired using Image Lab Software (BioRad). Relative intensity was quantified by densitometric analysis using ImageJ software.

Oxyblot Analysis of recombinant HIF-2α oxidation

To independently validate direct LOX-mediated oxidation, protein carbonylation was assessed using the OxyBlot Protein Oxidation Detection Kit (Sigma-Aldrich, Cat. no. S7150) with bacterially expressed recombinant HIF-2α. Purified recombinant HIF-2α was incubated with recombinant human LOX (Origene, Cat. no.TP313323) in oxidation buffer at 37°C for 45 min in the presence or absence of LOX inhibitor. Proteins were derivatized with 2,4-dinitrophenylhydrazine (DNPH) according to the manufacturer's instructions, resolved by SDS-PAGE, transferred to PVDF membranes, and carbonylated proteins were detected using anti-DNP antibody. This assay was used as an orthogonal validation of LOX-dependent oxidation in a cell-free system.

bioRxiv preprint doi: https://doi.org/10.64898/2026.07.24.739825; this version posted July 27, 2026. The copyright holder for this preprint (which was not certified by peer review) is the author/funder, who has granted bioRxiv a license to display the preprint in perpetuity. It is made available under a CC-BY 4.0 International license.

SUPPLEMENTARY FIGURES

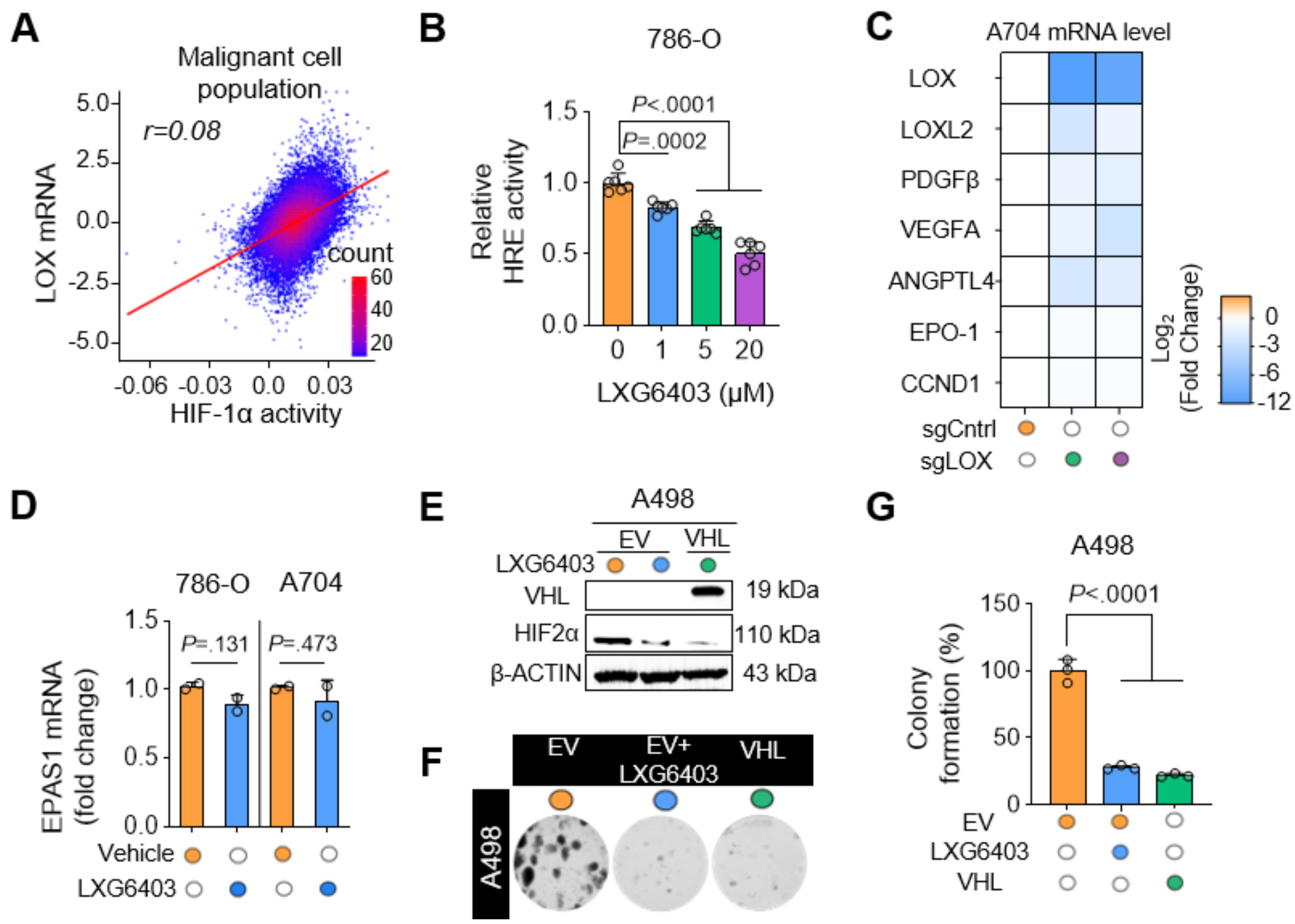

Supplementary Figure 1. LOX inhibition reduces HIF-2α transcriptional activity without affecting *EPAS1* mRNA expression. (A) Relationship between LOX expression and HIF-1α activity in malignant RCC cells. Scatterplot showing latent-log expression of LOX (x-axis) versus HIF-1α activity (y-axis). The line represents a linear model fit, and Pearson correlation values are indicated. **(B)** HRE-GFP reporter assay showing dose-dependent reduction of HIF-2α transcriptional activity upon LOX inhibition in 786-O cells (n = 6 technical replicates). **(C)** qRT-PCR analysis of canonical HIF-2α targets (LOX, LOXL2, PDGFB, VEGF, ANGPTL4, EPO-1, CCND1) in A704 sgLOX cells (n = 2). **(D)** qRT-PCR analysis of *EPAS1* mRNA showing unchanged transcript levels following LXG6403 treatment, indicating post-translational regulation of HIF-2α (n = 2). **(E)** Western blot analysis of VHL and HIF-2α in A498 cells transfected with

bioRxiv preprint doi: https://doi.org/10.64898/2026.07.24.739825; this version posted July 27, 2026. The copyright holder for this preprint (which was not certified by peer review) is the author/funder, who has granted bioRxiv a license to display the preprint in perpetuity. It is made available under a CC-BY 4.0 International license.

empty vector (EV) ± 7.5 μM LXG6403 or with wild-type VHL. β-ACTIN was used as a loading control here and in all immunoblots unless otherwise indicated. **(F)** Colony formation assay comparing A498 EV ± 7.5 μM LXG6403 and A498 cells expressing wild-type VHL. **(G)** Quantification of (F) (n = 3). Data are shown as mean ± SD. A two-sided Student's t-test was used to calculate the statistical difference between the two groups (D). One-way ANOVA with Dunnett's test was performed to compare more than one group (B, G). *P*-values are indicated. Colors denote experimental groups and are used consistently across graphs and representative images throughout the manuscript unless otherwise indicated. Open circles indicate control conditions, whereas filled circles indicate experimental conditions.

bioRxiv preprint doi: https://doi.org/10.64898/2026.07.24.739825; this version posted July 27, 2026. The copyright holder for this preprint (which was not certified by peer review) is the author/funder, who has granted bioRxiv a license to display the preprint in perpetuity. It is made available under a CC-BY 4.0 International license.

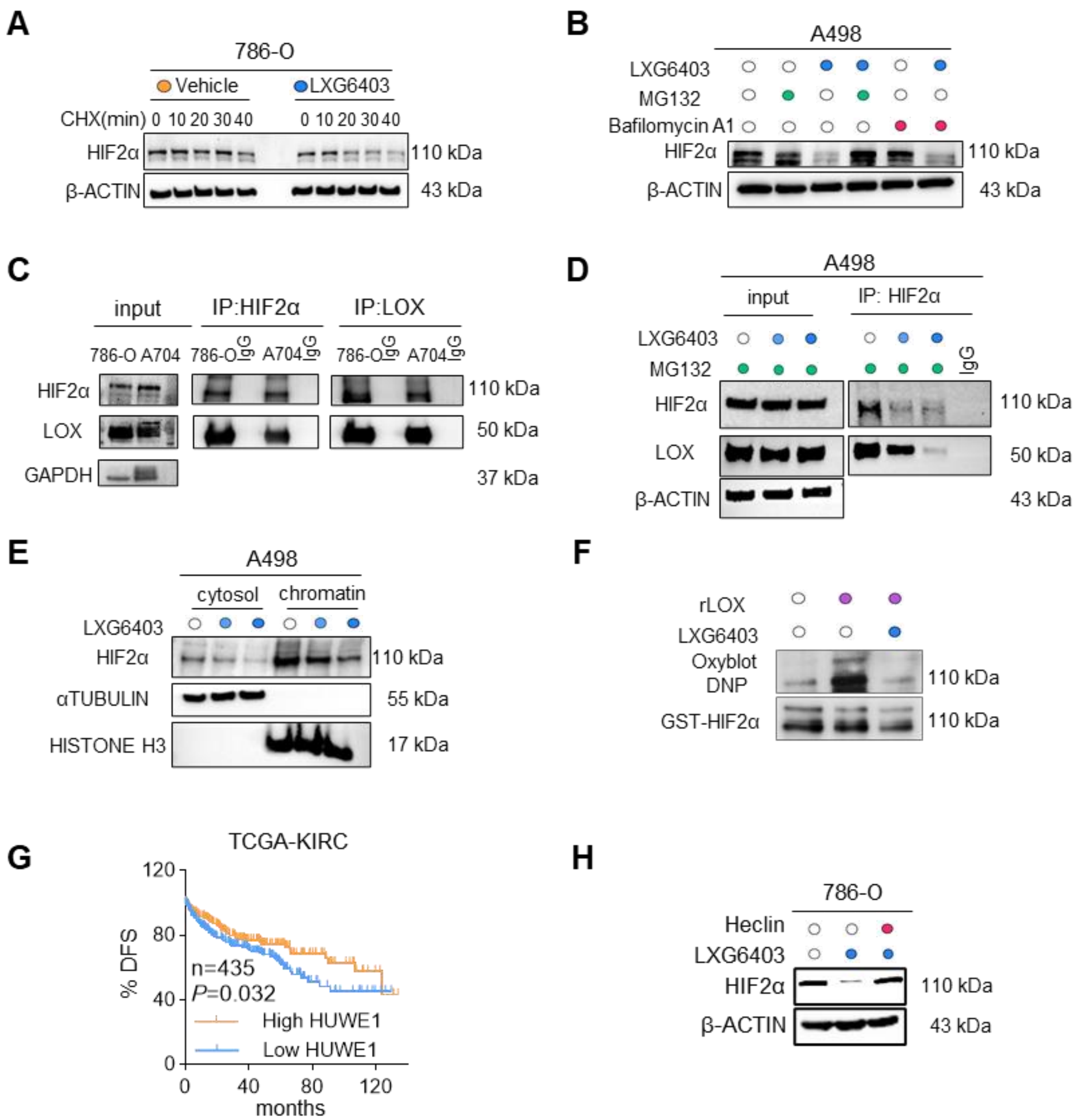

Supplementary Figure 2. LOX stabilizes HIF-2α and LOX inhibition induces HUWE1-mediated proteasomal degradation of HIF-2α, resulting in loss of its chromatin occupancy. (A) Representative immunoblot from cycloheximide (CHX) chase assay showing accelerated HIF-2α degradation upon LOX inhibition. β-ACTIN was used as a loading control here and in all immunoblotting figures, unless otherwise indicated. **(B)** Western blot of 786-O cells treated with ± LXG6403 and ± MG132 or bafilomycin A1, showing that proteasome inhibition rescues HIF-2α

bioRxiv preprint doi: https://doi.org/10.64898/2026.07.24.739825; this version posted July 27, 2026. The copyright holder for this preprint (which was not certified by peer review) is the author/funder, who has granted bioRxiv a license to display the preprint in perpetuity. It is made available under a CC-BY 4.0 International license.

abundance under LOX inhibition, whereas lysosomal inhibition does not (LXG6403 30μM for 24 hours, MG132 10μM for 6 hours, Bafilomycin A1 50 nM for 6 hours). **(C)** Co-immunoprecipitation (co-IP) and reverse-IP demonstrating a physical interaction between LOX and HIF-2α. GAPDH is used as a loading control. **(D)** Immunoblot of LOX and HIF-2α in A498 cells treated with LXG6403 for 24 hours in the presence of MG132 (10μM for the last 6 hours). Increasing blue color intensity corresponds to increasing LXG6403 concentrations (15 and 30 μM). **(E)** Chromatin fractionation assays showing reduced chromatin-bound HIF-2α upon LOX inhibition in a dose-dependent manner. Increasing blue color intensity corresponds to increasing LXG6403 concentrations (15 and 30 μM) for 48 hours. α-tubulin and Lamin B1 serve as fractionation control. **(F)** OxyBlot analysis of a bacterial cell-free system demonstrating LOX-mediated oxidation of recombinant HIF-2α. Incubation with recombinant LOX (rLOX) induces HIF-2α carbonylation, which is suppressed by LOX inhibitor treatment, LXG6403 (30μM for 2 hours). **(G)** Kaplan-Meier disease-free survival analysis based on HUWE1 expression in ccRCC patients from TCGA (n = 435). **(H)** Immunoblot analysis showing that inhibition of HECT-domain E3 ligases with heclin rescues HIF-2α protein levels following LOX inhibition, implicating HECT-type ligases in LOX-dependent HIF-2α turnover (LXG6403 30μM for 4 hours; Heclin 20μM for 30 minutes). Survival differences were evaluated using the log-rank (Mantel-Cox) test (G). *P*-values are indicated.

bioRxiv preprint doi: https://doi.org/10.64898/2026.07.24.739825; this version posted July 27, 2026. The copyright holder for this preprint (which was not certified by peer review) is the author/funder, who has granted bioRxiv a license to display the preprint in perpetuity. It is made available under a CC-BY 4.0 International license.

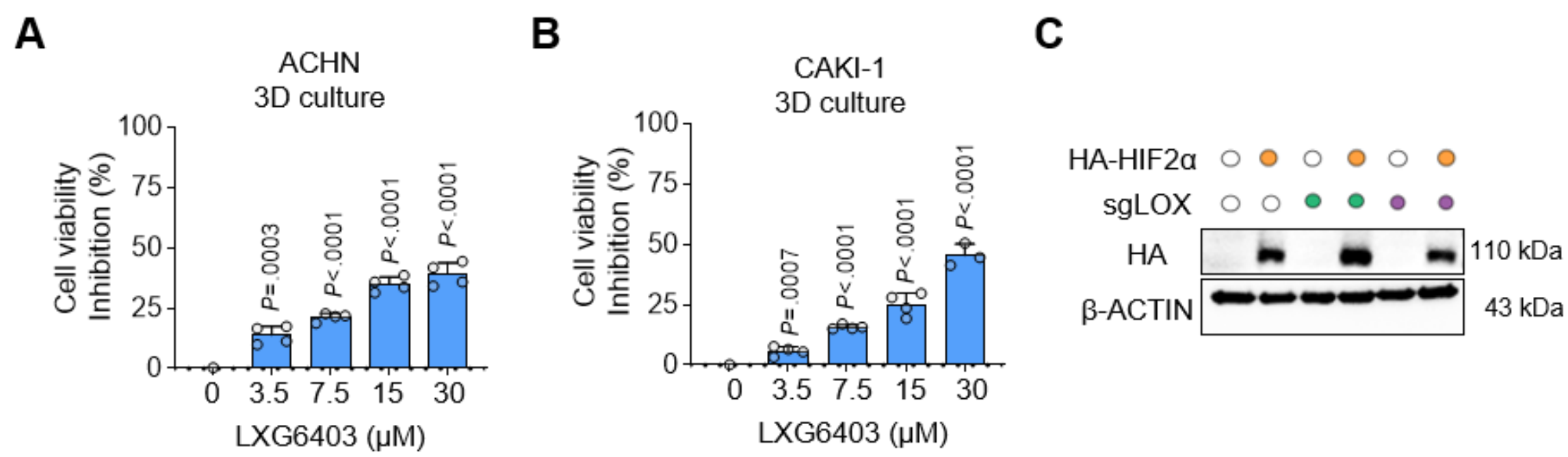

Supplementary Figure 3. LOX inhibition reduces 3D cell viability in VHL-wildtype ccRCC. (A, B) Dose-dependent reduction of 3D viability with LXG6403 treatment in *VHL*-wildtype cells ACHN (A) and CAKI-1 (B) (n = 4 technical replicates). **(C)** Immunoblot validating HA-HIF-2α overexpression in A704 sgLOX cells. Data are shown as mean ± SD. One-way ANOVA with Dunnett's test was performed to compare more than one group (A, B). *P*-values are indicated.

bioRxiv preprint doi: https://doi.org/10.64898/2026.07.24.739825; this version posted July 27, 2026. The copyright holder for this preprint (which was not certified by peer review) is the author/funder, who has granted bioRxiv a license to display the preprint in perpetuity. It is made available under a CC-BY 4.0 International license.

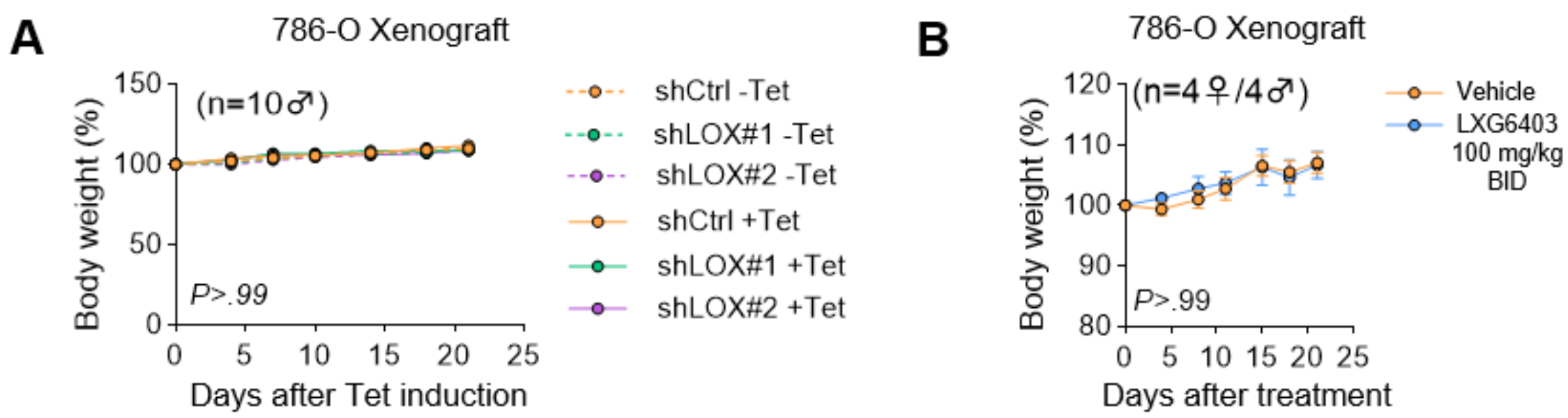

Supplementary Figure 4. LOX inhibition does not cause toxicity in vivo. (A, B) Body weight curves for NSG mice bearing 786-O xenografts with tetracycline-inducible shLOX or control during tetracycline administration (drinking water supplemented with 50 g/L sucrose and 1 g/L tetracycline) (n = 10 mice) (A), and in a sex-balanced 786-O xenograft cohort (n = 4 females, 4 males) treated with vehicle or LXG6403 (100 mg/kg, BID, p.o.) for 21 days (n = 8 NSG mice per group) (B). Data are shown as mean ± SEM. Statistical significance was determined by multiple comparisons with Holm's correction (A, B). *P*-values are indicated.

bioRxiv preprint doi: https://doi.org/10.64898/2026.07.24.739825; this version posted July 27, 2026. The copyright holder for this preprint (which was not certified by peer review) is the author/funder, who has granted bioRxiv a license to display the preprint in perpetuity. It is made available under a CC-BY 4.0 International license.

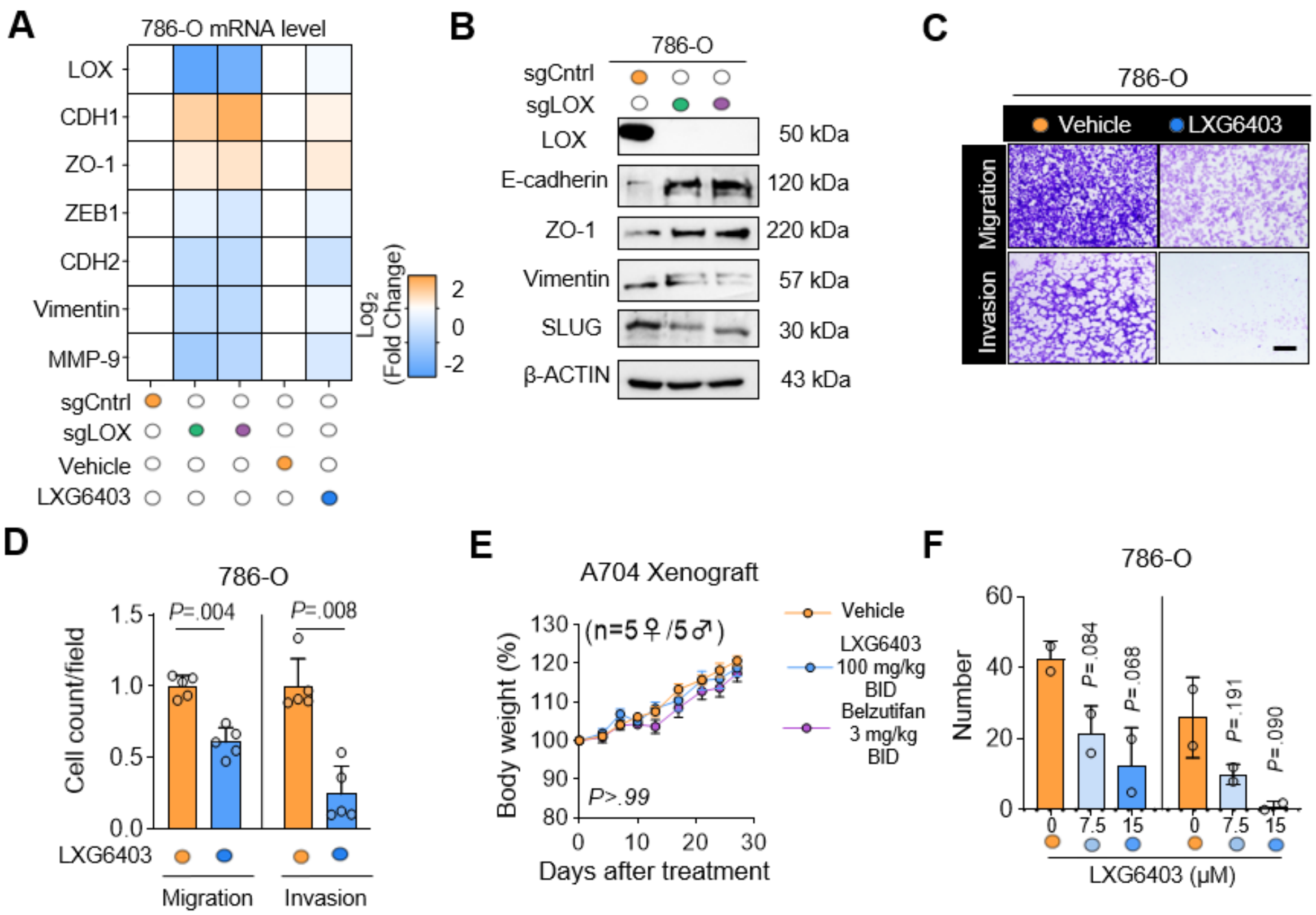

Supplementary Figure 5. Genetic or pharmacologic inhibition of LOX induces MET-like transition in VHL-mutant ccRCC. (A) Heatmap of qRT-PCR analysis of EMT marker changes upon siRNA-mediated LOX knockdown in 786-O cells (n = 2). **(B)** Immunoblot confirming EMT marker changes in sgLOX-expressing 786-O cells. **(C, D)** Transwell migration and invasion assays showing impaired motility in 786-O cells after LOX inhibition (C) and quantification (D) (n = 5 different images). **(E)** Body weight change of mice, showing no significant treatment-related toxicity (n = 5 female/5male NSG mice). **(F)** Quantification of spheres/sub-spheres number in 786-O cells treated with LXG6403 (7.5 and 15 μM) for 21 days (n = 2). Data are shown as mean ± SD from biological experiments. In vivo data are shown as mean ± SEM. Statistical significance was determined by unpaired two-sided Student's t-test between the two groups (D, F) and multiple comparisons with Tukey's and Holm's correction for in vivo data (E). *P*-values are indicated.

bioRxiv preprint doi: https://doi.org/10.64898/2026.07.24.739825; this version posted July 27, 2026. The copyright holder for this preprint (which was not certified by peer review) is the author/funder, who has granted bioRxiv a license to display the preprint in perpetuity. It is made available under a CC-BY 4.0 International license.

A

P-value				
Conditions	Junctions	Nodes	Segments	Branches
Basal media	ref.	ref.	ref.	ref.
Growth media	.025	.007	<.0001	.02
Basal media+ LOX ORF	.004	<.0001	<.0001	.0006

C

P-value				
Conditions	Junctions	Nodes	Segments	Branches
Basal media	.026	0.0016	.004	.002
Basal media+ FGF	.575	.0006	.009	.465
Growth media	ref.	ref.	ref.	ref.
Growth media+ axitinib	.054	.0009	.009	.013
Growth media+ LXG6403	.035	.00034	.005	.0032
Growth media+ combo	.025	.00019	.005	.009

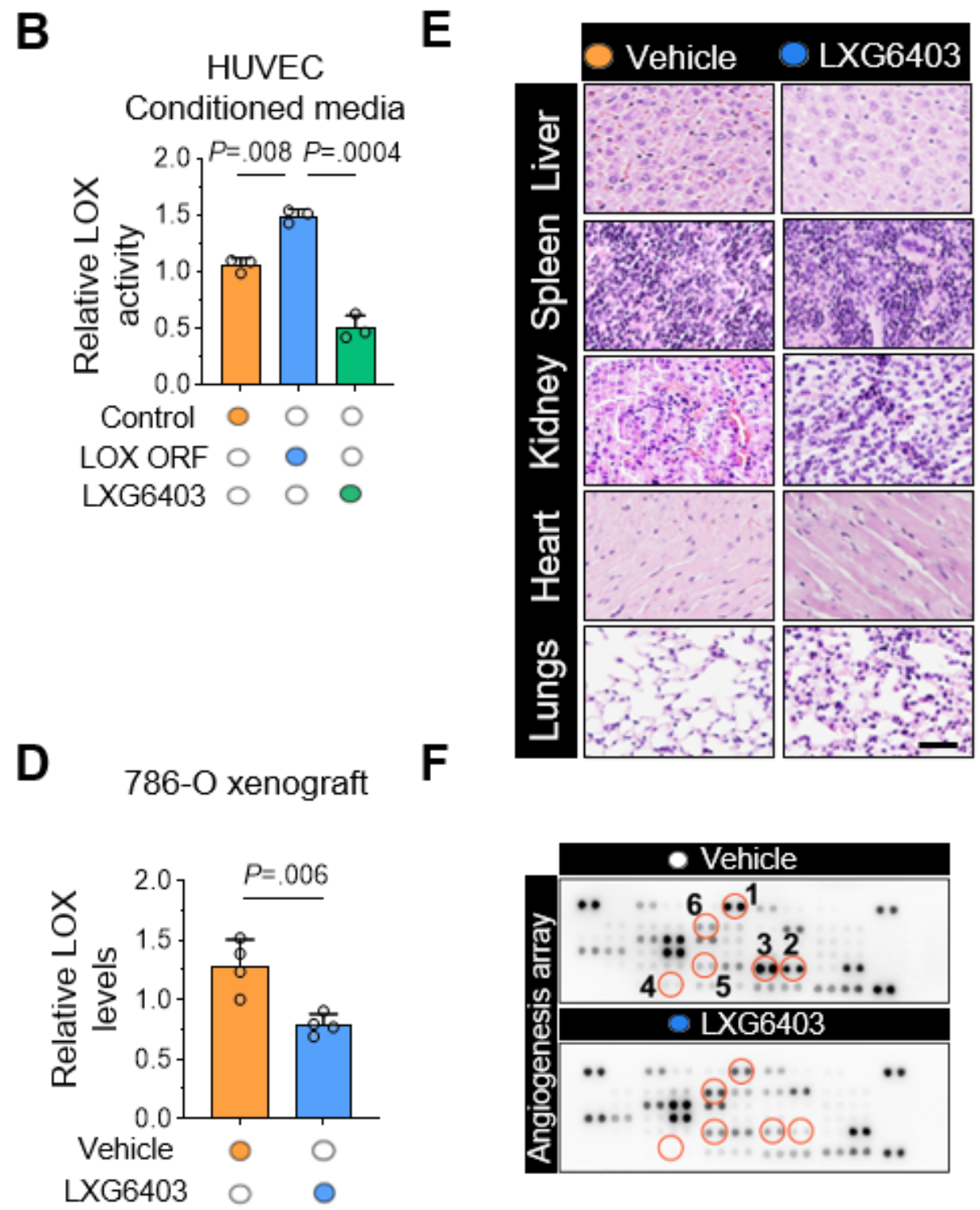

Supplementary Figure 6. LOX regulates angiogenic activity and hypoxic signaling in ccRCC models. (A) Statistical summary reporting *P*-values for HUVEC tubule formation metrics (junctions, nodes, segments, branches) across indicated conditions (Basal media, Growth media, and Basal media+ LOX-conditioned media (CM)). *P*-values derived from one-way ANOVA with Dunnett's (vs control) and Tukey's (all pairwise) post hoc tests. **(B)** Relative LOX activity in HUVEC conditioned media (n = 3 technical replicates) **(C)** Statistical summary reporting *P*-values for HUVEC tubule formation metrics (junctions, nodes, segments, branches) across indicated conditions (Basal media, Basal media with Fibroblast growth factor (FGF), Growth media with axitinib, Growth media with LXG6403, and Growth media with combination). *P*-values derived from one-way ANOVA with Dunnett's (vs control) and Tukey's (all pairwise) post hoc tests. **(D)** Quantification of serum LOX levels in NSG mice treated with vehicle and LXG6403 (100 mg/kg,

bioRxiv preprint doi: https://doi.org/10.64898/2026.07.24.739825; this version posted July 27, 2026. The copyright holder for this preprint (which was not certified by peer review) is the author/funder, who has granted bioRxiv a license to display the preprint in perpetuity. It is made available under a CC-BY 4.0 International license.

p.o, 8h) (n = 4 mice). **(E)** H&E staining of non-tumor organs showed no detectable histopathological abnormalities following LXG6403 treatment (n = 4 mice). **(F)** Angiogenic protein array blot (1: Angiopoietin-1, 2: PDGF-AA, 3: PDGF-AB/-BB, 4: VEGF, 5: Endostatin, 6: Platelet factor 4), (n = 2 mice). Data are shown as mean ± SD. Statistical significance was determined by unpaired two-sided Student's t-test between the two groups (B, D). *P*-values are indicated.

bioRxiv preprint doi: https://doi.org/10.64898/2026.07.24.739825; this version posted July 27, 2026. The copyright holder for this preprint (which was not certified by peer review) is the author/funder, who has granted bioRxiv a license to display the preprint in perpetuity. It is made available under a CC-BY 4.0 International license.

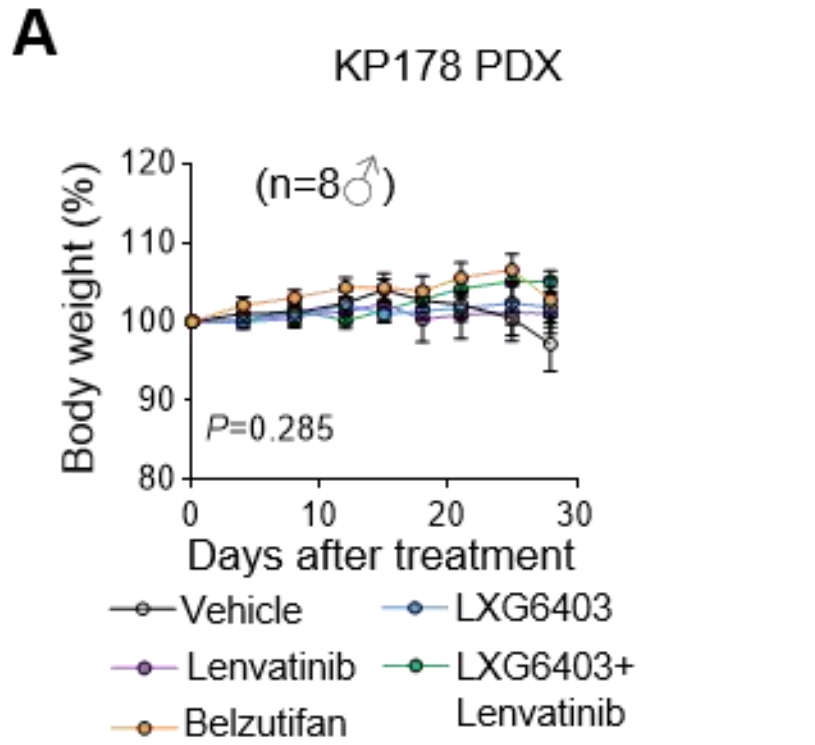

B

CBC analysis	KP178 PDX				
Parameters (mean)	Vehicle (n=4)	Lenvatinib (n=4)	Belzutifan (n=4)	LXG6403 (n=4)	LXG6403 + Lenvatinib (n=4)
RBC (10^{12} cells/l)	8.6	8.2	7.9	7.5	8.0
HGB (g/dl)	9.3	9.3	9.9	9.5	9.4
HCT (%)	41.8	38.8	37.6	35.2	38.8
Platelets (10^9cells/l)	516.8	755.5	459.5	323.8	440.0
WBC (10^9cells/l)	1.1	1.2	1.3	1.3	1.2
P-values: not significant (ns) for all parameters compared to combo					

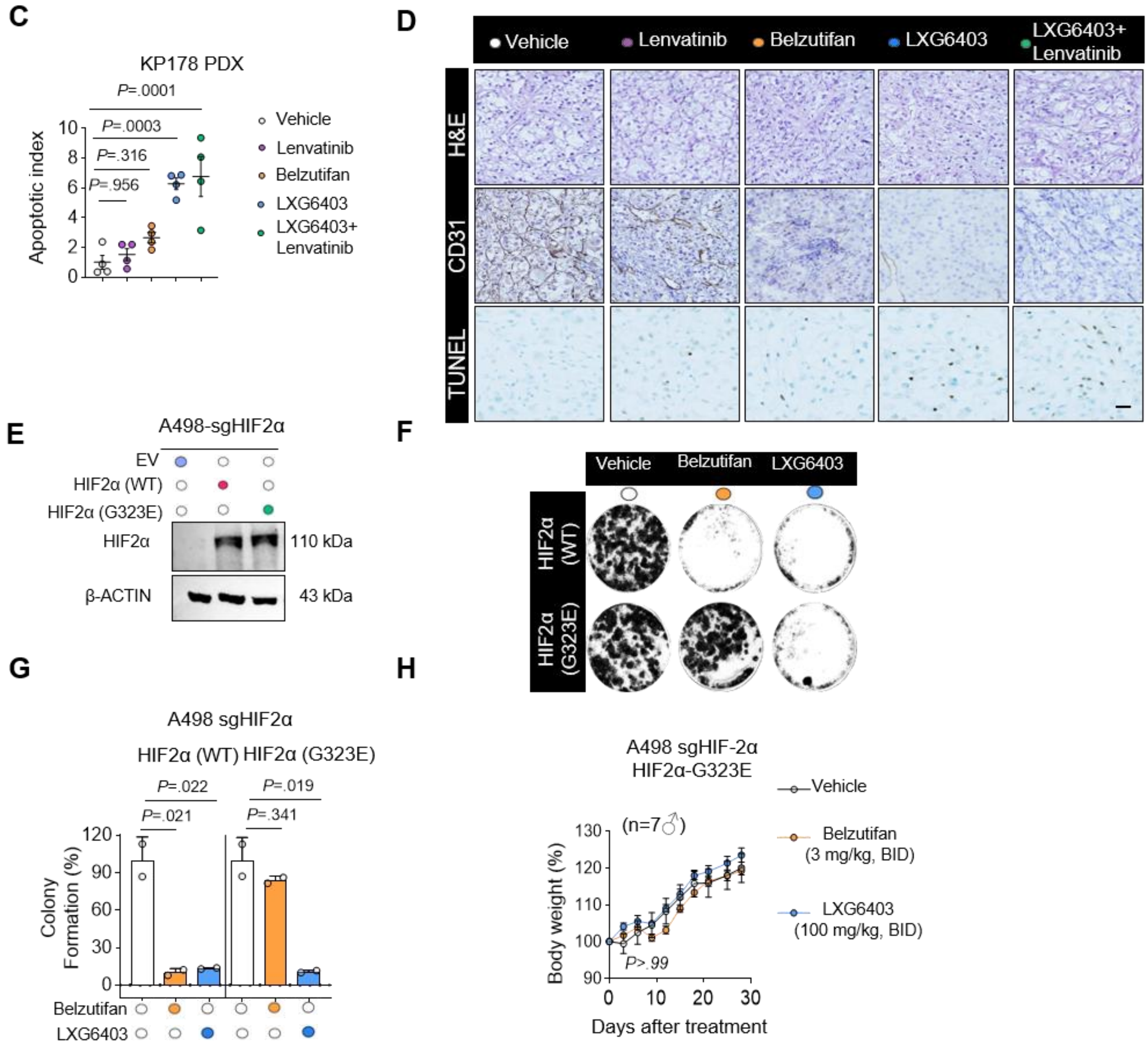

bioRxiv preprint doi: https://doi.org/10.64898/2026.07.24.739825; this version posted July 27, 2026. The copyright holder for this preprint (which was not certified by peer review) is the author/funder, who has granted bioRxiv a license to display the preprint in perpetuity. It is made available under a CC-BY 4.0 International license.

Supplementary Figure 7. LOX inhibition in combination with lenvatinib does not cause toxicity while inducing apoptosis, and the validation of HIF-2α expression upon wildtype or G323E mutant HIF-2α overexpression. (A) Percent body weight change in the KP78 PDX study: vehicle, lenvatinib (10 mg/kg, QD, p.o), LXG6403 (75 mg/kg, BID, p.o.), and combination for 28 days (n = 8 male NSG mice) and immunoblot from KP178 ccRCC PDX tumor lysate showing high levels of LOX and VEGFA, β-actin as loading control. **(B)** Complete blood counts (RBC, HGB, HCT, WBC, PLT) from KP178 PDX study demonstrating no hematologic toxicity (n = 4 mice). **(C)** Quantification of apoptotic index from TUNEL-stained tumor sections from (A) (n = 4 tumors). **(D)** Representative images of H&E, CD31 and TUNEL staining in tumor sections from (A) (n = 4 tumors). **(E)** Immunoblot showing overexpression of HIF-2α wildtype (WT) and HIF-2α G323E in sgHIF-2α-expressing A498 cells. **(F)** Colony formation assay showing reduced clonogenic potential upon LOX (15 μM) or belzutifan (1μM) treatment in sgHIF-2α-expressing A498 cells overexpressed either with HIF-2α WT or HIF-2α G323E (n = 2). **(G)** Quantification of (F). **(H)** Percent body weight change in A498 sgHIF-2α HIF-2α-G323E xenograft cohort treated with vehicle, belzutifan (3 mg/kg, BID, p.o.), or LXG6403 (100 mg/kg, BID, p.o.) for 28 days (n = 7 male mice). Data are shown as mean ± SD from biological experiments. In vivo data are shown as mean ± SEM. Statistical significance was determined by unpaired two-sided Student's T-test (B) or two-way ANOVA with Dunett's (C, G), and Holm's (A, H) corrections. *P*-values are indicated.

bioRxiv preprint doi: https://doi.org/10.64898/2026.07.24.739825; this version posted July 27, 2026. The copyright holder for this preprint (which was not certified by peer review) is the author/funder, who has granted bioRxiv a license to display the preprint in perpetuity. It is made available under a CC-BY 4.0 International license.

SUPPLEMENTARY REFERENCES

1. Mishra, R.R., *et al.* Reactivation of cAMP Pathway by PDE4D Inhibition Represents a Novel Druggable Axis for Overcoming Tamoxifen Resistance in ER-positive Breast Cancer. *Clin Cancer Res* **24**, 1987-2001 (2018).
2. Assidicky, R., *et al.* Targeting HIF1-alpha/miR-326/ITGA5 axis potentiates chemotherapy response in triple-negative breast cancer. *Breast Cancer Res Treat* **193**, 331-348 (2022).
3. Carpentier, G., *et al.* Angiogenesis Analyzer for ImageJ - A comparative morphometric analysis of "Endothelial Tube Formation Assay" and "Fibrin Bead Assay". *Sci Rep* **10**, 11568 (2020).
4. Saatci, O., *et al.* Targeting lysyl oxidase (LOX) overcomes chemotherapy resistance in triple negative breast cancer. *Nat Commun* **11**, 2416 (2020).

bioRxiv preprint doi: https://doi.org/10.64898/2026.07.24.739825; this version posted July 27, 2026. The copyright holder for this preprint (which was not certified by peer review) is the author/funder, who has granted bioRxiv a license to display the preprint in perpetuity. It is made available under a CC-BY 4.0 International license.

Supplementary Table 1. Clinicopathologic characteristics of the ccRCC tissue microarrays (TMAs)

**Entire TMA Cohort, n**	**204**
**Histologic Grade, n (%)**	
I	16 (7.8%)
II	90 (44.1%)
III	83 (40.7%)
IV	15 (7.4%)
Clinical subset, n*	65 out of 204
**Age, Years, Median (Range)**	60 (23-87)
**Sex, n (%)**	
Male	44 (67.7%)
Female	21 (32.3%)
**Pathologic T Stage, n (%)**	
pT1	38 (58.5%)
pT2	0 (0%)
pT3	26 (40%)
pT4	1 (1.5%)

*Clinical annotations (age, sex, and pathologic t stage) were available for 65 of the 204 patients included in the TMA cohort. The remaining cases were reported previously[61].

bioRxiv preprint doi: https://doi.org/10.64898/2026.07.24.739825; this version posted July 27, 2026. The copyright holder for this preprint (which was not certified by peer review) is the author/funder, who has granted bioRxiv a license to display the preprint in perpetuity. It is made available under a CC-BY 4.0 International license.

Supplementary Table 2. List of antibodies used in WB, IP, and IF

List of antibodies used in Western blot (WB), Proximity ligation assay (PLA), immunohistochemistry (IHC), immunoprecipitation (IP) and chromatin immunoprecipitation (ChIP) experiments.

Antibody	Provider	Catalog number	WB dilution	IP dilution	PLA/IHC/ChIP dilution
LOX	Sigma-Aldrich	L4794 (RRID:AB_1840993)	1:1000	1:250	1:100 (PLA), 1:600 (IHC)
HIF-2α	Novus	NB100-122 (RRID:AB_10002593)	1:1000	1:100	1:600 (IHC), 1:100 (ChIP)
HIF-2α	Santa Cruz	sc-46691 (RRID:AB_627523)			1:100 (PLA)
HA	Invitrogen	26183 (RRID:AB_2533049)	1:7500		-
FLAG	Proteintech	80010-1-RR (RRID:AB_2882940)	1:7500	1:250	-
β-ACTIN	Sigma	A2228 (RRID:AB_476697)	1:10000	-	-
UBIQUITIN	Cell Signaling	58395 (RRID:AB_3075532)	1:2000	-	-
STREPTAVIDIN-HRP	Thermo Fischer	EMDA5441 (RRID:AB_2619743)	1:7500	-	-
VHL	Cell signaling	68547 (RRID:AB_271627)	1:1000	-	-
E-CADHERIN	BD Biosciences	BD610181 (RRID:AB_397580)	1:1000	-	-
ZO-1	Cell signaling	5406 (RRID:AB_1904187)	1:1000	-	-
VIMENTIN	Cell signaling	5741 (RRID:AB_10695459)	1:1000	-	-
ZEB-1	Santa Cruz	sc-515797 (RRID:AB_2934316)	1:1000	-	-
SLUG	Abcam	ab27568 (RRID:AB_777968)	1:1000	-	-
GAPDH	Cell signaling	2118 (RRID:AB_561053)	1:1000	-	-
α-TUBULIN	Santa Cruz	sc-32293 (RRID:AB_628412)	1:1000	-	-
LAMIN B1	Santa Cruz	sc-374015 (RRID:AB_10947408)	1:1000	-	-
PDGFβ	Cell Signaling	3169 (RRID:AB_2162497)	1:1000	-	-
p-PDGFβ	Cell Signaling	3166 (RRID:AB_331365)	1:1000	-	-
FGFR1	Cell Signaling	9740 (RRID:AB_11178519)	1:1000	-	-
p-FGFR1	Cell Signaling	52928 (RRID:AB_2799424)	1:1000	-	-
VEGFR2	Cell Signaling	2472 (RRID:AB_33111)	1:1000	-	-
p-VEGFR2	Cell Signaling	2478 (RRID:AB_331377)	1:1000	-	-
ANG-1	Proteintech	27093-1-AP (RRID:AB_3085925)	1:1000	-	-
VEGFA	Cell Signaling	50661(RRID: AB_3751965)	1:1000	-	-

bioRxiv preprint doi: https://doi.org/10.64898/2026.07.24.739825; this version posted July 27, 2026. The copyright holder for this preprint (which was not certified by peer review) is the author/funder, who has granted bioRxiv a license to display the preprint in perpetuity. It is made available under a CC-BY 4.0 International license.

SRC	Cell Signaling	2109 (RRID:AB_2106059)	1:1000	-	-
p-SRC	Cell Signaling	2101 (RRID:AB_331697	1:1000	-	-
FAK	Cell Signaling	3285 (RRID:AB_2269034	1:1000	-	-
AKT	Santa Cruz	9272 (RRID:AB_329827)	1:1000	-	-
p-AKT	Cell Signaling	13038 (RRID:AB_2629447)	1:1000	-	-
HRP-LINKED ANTI-MOUSE IGG	Cell Signaling	7076 (RRID:AB_330924)	1:5000	-	-
HRP-LINKED ANTI-RABBIT IGG	Cell Signaling	7074 (RRID:AB_2099233)	1:5000	-	-

Supplementary Table 3. Primer sequences used in qRT-PCR.

Sequences of qRT-PCR primers	
**Primer**	**Sequence (5'-3')**
**LOX forward primer**	GGATACGGCACTGGCTACTT
**LOX reverse primer**	GACGCCTGGATGTAGTAGGG
**LOXL2 forward primer**	TGACTGCAAGCACACGGAGGAT
**LOXL2 reverse primer**	TCCGAATGTCCTCCACCTGGAT
**PDGFβ forward primer**	CTCGATCCGCTCCTTTGATGA
**PDGFβ reverse primer**	CGTTGGTGCGGTCTATGAG
**VEGFA forward primer**	TTGCCTTGCTGCTCTACCTCCA
**VEGFA reverse primer**	GATGGCAGTAGCTGCGCTGATA
**ANGPTL4 forward primer**	GGCTCAGTGGACTTCAACCG
**ANGPTL4 reverse primer**	CCGTGATGCTATGCACCTTCT
**EPO1 forward primer**	TTCTGTGCTTCACCGAGCGGTT
**EPO1 reverse primer**	CGACACAGCTTCCATGGCTCAT
**CCND1 forward primer**	GCTGCGAAGTGGAAACCATC
**CCND1 reverse primer**	CCTCCTTCTGCACACATTTGAA
**CDH1 forward primer**	CAGGAACACAGGAGTCATC
**CDH1 reverse primer**	CAAAATCCTCCCTGTCC
**ZO1 forward primer**	GTCCAGAATCTCGGAAAAGTGCC
**ZO1 reverse primer**	CTTTCAGCGCACCATACCAACC
**ZEB1 forward primer**	GGCATACACCTACTCAACTACGG
**ZEB1 reverse primer**	TGGGCGGTGTAGAATCAGAGTC
**CDH2 forward primer**	CCTCCAGAGTTTACTGCCATGAC
**CDH2 reverse primer**	GTAGGATCTCCGCCACTGATTC
**Vimentin forward primer**	TGTCCAAATCGATGTGGATGTTTC
**Vimentin reverse primer**	TTGTACCATTCTTCTGCCTCCTG

bioRxiv preprint doi: https://doi.org/10.64898/2026.07.24.739825; this version posted July 27, 2026. The copyright holder for this preprint (which was not certified by peer review) is the author/funder, who has granted bioRxiv a license to display the preprint in perpetuity. It is made available under a CC-BY 4.0 International license.

**MMP9 forward primer**	GCCACTACTGTGCCTTTGAGTC
**MMP9 reverse primer**	CCCTCAGAGAATCGCCAGTACT
**ACTB forward primer**	CCAACCGCGAGAAGATGA
**ACTB reverse primer**	CCAGAGGCGTACAGGGATAG
**HPRT forward primer**	TGACCTTGATTTATTTTGCATACC
**HPRT reverse primer**	CGAGCAAGACGTTCAGTCCT

Supplementary Table 4. siRNA and sgRNA sequences.

Sequences of sgRNAs and siRNAs used	
**Primer**	**Sequence**
**sgLOX-#1 forward**	GCTTGGTGGAGACTGAGATAC
**sgLOX-#1 reverse**	CAGGTCTGGGCCTTTCATAA
**sgLOX-#2 forward**	GCAGATCCAATGGGAGAACA
**sgLOX-#2 reverse**	AGCAGGTCATAGTGGCTAAAC
**siRNA LOX-#1**<br>Dharmacon, Cat. no. D-009810-04-0005	CACAGGACAUCAUGCGUAU
**siRNA LOX-#2**<br>Dharmacon, Cat. no. D-009810-17-0005	CAAUUUCACCGUAUUAGAA

Supplementary Table 5. ChIP-qPCR primers.

Sequences of ChIP qPCR primers	
**Primer**	**Sequence (5'-3')**
**VEGFA forward**	CACAGACCTTCACAGCCATC
**VEGFA reverse**	CCCAGCGTAGACAGTTGAGT
**SERPINE1 forward**	GGAAAAGCAGGCAACGTGAG
**SERPINE1 reverse**	TGTCTGTCTCTCCCGGATGT
**PTHrP forward**	CTCTTTGCGACTAGCTCACTT
**PTHrP reverse**	GAGAACAAGTTTCAAGTGCG
**CCND1 forward**	ATGACCCTCAAAAGCCCAGA
**CCND1 reverse**	GGTTAGCGAGCGTAAAGAGC
